# Supplementary material for: ArborSim: Articulated, branching, OpenSim routing for constructing models of multi-jointed appendages with complex muscle-tendon architecture
Source: PLoS Comput Biol. 2024 Jul 5;20(7):e1012243. doi: 10.1371/journal.pcbi.1012243 (PMC11253963; doi:10.1371/journal.pcbi.1012243)
Supplement: Attachment — Submitted filename: ArborSim_PLOS_Cmputational_Biology_ResponseToReviewers.pdf [file pcbi.1012243.s004.pdf]

`ArborSim`: Articulated, branching, OpenSim routing for constructing models of multi-jointed appendages with complex muscle-tendon architecture

Response to Reviewers

Xun Fu, Jack Withers, Juri A. Miyamae, Talia Y. Moore

April 28, 2024

We would like to start by expressing our gratitude to the reviewers for their thorough review of our paper. The comments provided were extremely helpful and insightful. Following the recommendations from both the reviewers and the editors, we have made several modifications to the paper. These changes are summarized in detail below and a highlighted manuscript is attached. In the modified manuscript, new text to address comments is in blue, removed text is in ~~red~~ and stricken out, and the corresponding reviewer's comment is referenced in the format 'reviewer#.comment' in the left margin. In the interest of keeping the response statement short, we have not copied, verbatim, the changes that are made in the paper. Figures and tables that appear only in the response letter are denoted with the prefix "R" before the number.

Reviewer 1

Comment 1 :

This is a solid contribution. It will accelerate the workflow for researchers using OpenSim to model more complex musculoskeletal structures. There is much recent interest in tails, but the extension to fingers and other appendages makes it broadly useful. The text is very clearly written and mostly easy to follow. A couple minor additions would make it a very useful contribution.

We thank the reviewer for their comments. #1.1

Comment 2 :

The toy model is appropriate at this stage, but it will be nice to see this applied to in vivo data. Getting measurements with which to validate the more refined approach will be challenging, but a nod to this exciting prospect could be made in the discussion?

We thank the reviewer for their insightful comments. We agree that obtaining *in vivo* measurements to validate the proposed branching modeling approach is challenging at the moment and would likely warrant another publication. We appreciate the encouragement to discuss this exciting prospect in the paper. Accordingly, a detailed exploration of prospective methods for validating the proposed approach, highlighting potential strategies has been included in the Discussion section. #1.2

Comment 3 :

Abstract: we found that the number of tendon branches and the number of joints between branches are most sensitive to branching modeling method. These things are the most sensitive amongst which pool of candidates? What other parameters/variables were studied?

We thank the reviewer for their comments. In the abstract, we have now clarified that our study assessed the impact of different branching modeling methods on three key parameters: the number of tendon branches, the number of joints between branches, and the ratio of muscle fiber length to muscle-tendon unit length. These three parameters were selected based on the variability observed in muscle-tendon branching architectures across different mammalian tails, as explained in the "Design and implementation: Comparative simulation case study" section of our paper. #1.3

Comment 4 :

Reading the author summary, I see that perhaps this is meant to drive home how important the modeling approach to handling branching is, and that `ArborSim` is good at this or presents a unified approach. Perhaps this should be more explicitly stated?

We appreciate the reviewer's suggestion to more explicitly state in our paper that a key takeaway is the importance of accurate branching modeling in biological multi-jointed appendages; `ArborSim` provides a unified framework that facilitates further detailed exploration in this field. In response, we have revised the author summary and the Conclusion section to highlight this aspect more clearly. #1.4

Comment 5 :

L3 in vivo italicized

We thank the reviewer for pointing out the term *in vivo* should be italicized. We have made this change throughout the paper #1.5

Comment 6 :

L73 - multiple independent compartment: so each tendon has it's own separate muscle, instead of all tendons converging on the same muscle?

Yes. In those existing musculoskeletal models of the human hand, to model the Extensor Digitorum Communis (EDC) muscle, each tendon associated with the EDC is assigned to connect to a portion of the muscle. These portions are modeled independently, assuming there are no physical interconnections or interactions between them. #1.6

Comment 7 :

The example in L82-L86 is convincing.

We thank the reviewer for their comment. #1.7

Comment 8 :

L124 Can the large differences be compared with any experimental data to determine which method is more accurate? Presumably the more realistic branched method?

We appreciate the reviewer's practical question and have elaborated more in the manuscript, including adding a new Figure 7. While we maintain that this empirical validation is outside the scope of our study, as this manuscript is limited to introducing a generalized model building framework without building a specific model, we recognize the importance of ensuring the possibility that a model can be validated in the future. To address this concern, we added two descriptions of how one would experimentally evaluate the accuracy of the branched model, both at the scale of a single branched tendon and at the whole joint scale. We plan to perform these analyses in a future study in which we model a specific anatomical feature.

This comment also led us to explore how the structural differences between the proposed and conventional methods for modeling branched MTUs would affect muscle contraction. To this end, we added the new Figure 7, which shows the muscle fiber length through time for the one branched MTU model (green) versus the many independent MTU models (gray). As shown in the Figure, there are large differences between the muscle fiber lengths when modeled independently with the conventional method, which would inaccurately predict the force and velocity of muscle contraction in a system with one muscle connected to multiple tendon branches. In contrast, our proposed branching modeling method inherently avoids this issue, coupling the muscles and tendons that within the same network. Therefore, we expect our proposed branching modeling method to provide more accurate results for systems in which one muscle is attached to multiple tendon insertions. #1.8

Comment 9 :

L340-L352. It's not clear what the function of the extra spherical body is at the branching point, and why it's necessary. Do the branched tendons stem off of it, like a manifold? Is it rigid, or made of ligament/tendon material? Or is the function of it to create a "wrapping surface" that splits the branches of the tendon apart?

We apologize for the lack of clarity. The extra spherical body is defined as a rigid body of infinitesimally small mass and size that can move relative to the vertebral bodies as the musculature contracts and extends. The function of the spherical body is to allow for the ligament elements to be attached to the MTU to create the branched structure. This is necessary because the muscle-tendon units previously generated by OpenSim could only include one muscle and one tendon per MTU. This constraint inherently prevented the modeling of complex branching muscle-tendon networks using a single unit. #1.9

Comment 10 :

What happens if this spherical body branching points slides onto a wrapping surface? Is that ok? Will they interact physically?

We appreciate the foresight of the reviewer for thinking of this important situation. OpenSim does not currently support direct interactions between wrapping surfaces and introduced rigid bodies, only interactions with muscle-tendon units and ligaments. However, OpenSim does include an element called "contact geometry" that does support interactions with rigid bodies. Contact geometries influence the motion of rigid bodies during interactions, functioning similarly to how wrapping surfaces interact with muscle-tendon units and ligaments, but specifically tailored for rigid bodies. Therefore, if a branched tendon is expected to interact with a wrapping surface, we suggest overlaying the wrapping surface with an identical contact geometry to ensure accurate contact dynamics. #1.10

Comment 11 :

Fig 6 is great, and shows the power of the architecture.

We thank the reviewer for their compliment. #1.11

Comment 12 :

L551-553 great argument for this work.

We thank the reviewer for their compliment.

Comment 13 : #1.12

Figure 7 caption - this needs to say what statistical test was run and what the sample size is, and what the sample population is. T-test, maybe? Are the replicates simulations with different levels of maximal isometric force over the range specified in the main text? Would be nice if the caption made the figure more "stand alone."

> We thank the reviewer for their constructive comment. In response, we have updated the captions for what were originally Figure 7 and Figure 8 (now Figures 8 and 9) to include detailed information about the statistical tests employed. We have also clearly stated the sampling conditions under which the data was collected to enhance the clarify and self-sufficiency of the figures. #1.13

Reviewer 2

Comment 1 :

This paper describes a novel software tool to integrate models with many joints containing complex muscle-tendon architectures into the existing OpenSim biomechanical modeling software. I believe that the work is novel and will be a useful tool for the scientific community that enables different types of biomechanical simulations to be performed. The manuscript is also well-written. However, there are a couple of points that should be addressed prior to publication.

> We thank the reviewer for their positive comments. We have provided detailed responses to each comment as follows. #2.1

Major concerns

Comment 2 :

The authors detail the difference between their novel muscle-tendon modeling approach compared with traditional OpenSim models with a couple of toy examples, demonstrating differences in resulting motion from identical muscle excitation, sensitivity to model error, and computation time. For the computational timing analysis, the authors present the average computational time across multiple models, but no standard deviations are given. This information should be added, with significance testing between conditions as in Figs 7-8.

> We thank the reviewer for their valuable comment. We have now included the variance of the computational times across different architectural categories in the table. Additionally, we have performed significance testing on the computational times between the two methods in the Results and discussion section, similar to what was presented in Figures 7 and 8. #2.2

Comment 3 :

When describing the sensitivity analysis, the authors mention that some of the simulations would fail when applying higher perturbations to ligament resting length. Was this true for the traditional modeling approach or only with the new approach?

We appreciate the reviewer's concern regarding the potential simulation failures. As stated in the methods section, we were able to overcome the simulation failures by adjusting our input parameters. In our initial simulations, failures occurred only when the contraction of the muscle-tendon unit caused the floating body to move beyond the two consecutive via points of the muscle-tendon units, which partially delineate the paths of the muscle-tendon units. This was likely because our toy examples included many via points to constrain the MTU paths. For models of real musculoskeletal systems, we instead recommend using contact geometries, which are elaborated in our response to Comment 10 from Reviewer 1, to guide the line of action for muscle-tendon units and ligaments. For the purposes of our study, because our toy models do not depict real biological systems, we opted to adjust the input parameters to avoid these issues. #2.3

Minor concerns

Comment 4 :

Figure 6 should have a legend describing the difference in colors

We have included a legend explaining the colors in Figure 6. #2.4

Comment 5 :

Line 421 – what does N.B. mean?

We apologize for the lack of clarity. N.B. stands for "Nota Bene", which is a Latin phrase that is used to emphasize an important point in a text. We changed this in the manuscript to "note that." #2.5

Comment 6 :

There are some minor inconsistencies in wording/punctuation in the manuscript that should be checked (ex. "the OpenSim" − > OpenSim in Line 379, "Opensim − > OpenSim" in Line 483)

We thank the reviewer's comment. We have further proofread the paper to address some inconsistencies in wording/punctuation in the paper. #2.6

Reviewer 3

Comment 1 :

Excellent idea. Developing detailed musculoskeletal model of animals especially ones other than human is very important and the spirit of this work is inline with doing so. The work presents a complete pipeline of going from modeling the mechanics to simulating them by adding to the existing infrastructure of OpenSim. Detailed and clear methodology section describing all the steps taken in the paper to (i) reproduce and (ii) reuse and (iii) reimplement if needed in other frameworks. Very good idea of using ligaments as tendons to model splitting in muscles.

We thank the reviewer for their comments. #3.1

Major concerns

Comment 2 :

No rigorous discussion on the observations from the experiments. In the results from the comparative simulations, tendon branch count and joint count show a non-linear relationship. This has been attributed to a complex, non-linear relationship in the branched muscle models. There needs to be a more in-depth analysis and reflection on where do these differences come from. Are they a result of initialization?

We thank the reviewer for their valuable comments. We have added further analysis to the paper that explains the differences in kinematics observed when using two distinct methods to model branched muscle-tendon architecture. Our findings reveal that the conventional method, which models the architecture with multiple parallel, independent muscle-tendon units, does not consistently achieve uniform muscle contraction as expected (see the new Figure 7). In contrast, our proposed method, which links muscles and tendons within the networks, circumvents this issue. #3.2

Comment 3 :

No plots or results from the muscle dynamics. Researchers would be interested to know why the kinematics are different between the presented approach and the conventional approach.

We thank the reviewer for their helpful comment. We have now included a plot in the paper that shows the changes in muscle fiber length over time (Figure 7). As noted in our response to Comment 1 and detailed in the main text, the differences in muscle fiber length changes explain the motion differences between the two methods. #3.3

Comment 4 :

How to validate the current methodology? Any comparisons to the ground truth? It is not enough to provide that there is a difference in kinematics when the models are switched. A stronger evidence needs to provided to prove that the presented mode is indeed better at capturing the dynamics of complex musculature. May be use existing OpenSim models with split tendons and compare with the proposed work?

We thank the reviewer for their comment. To address the validation of our method, we have added a discussion on validating our proposed branching modeling method, which includes a detailed outline of the necessary data collection required. While we agree that it is essential to describe exactly how to go about validating this kind of model, it is our view that matching empirical results from a specific tendon is outside the scope of our study. Such analysis will require a very different set of resources and is planned for a future study when we introduce a model of a specific biological system.

In terms of providing stronger evidence that our branching method is capable of more effectively capturing the dynamics of complex musculature, as previously addressed in responses to Comments 2 and 3, we have included additional analysis of muscle fiber length. This analysis highlights the shortcomings of traditional branching models, which do not adequately account for the physical interactions within branched muscle-tendon networks. In contrast, our method effectively integrates these interactions. Consequently, we believe this confirms the enhanced capability of our proposed method. #3.4

Comment 5 :

It is good that proposed method has the same/similar computational overhead as the conventional method. I was hoping for a code profile to clearly show what parts of the code spend the most time and thereby reflect on how future iterations of the methodology can improve it.

We thank the reviewer for their comment. In response, we have conducted profiling of both branching modeling methods. The results indicate that the most time-consuming aspect is the evaluation and integration of the forward dynamics within OpenSim, which advances the state of a system through time using differential equations.

This step is inherently affected by the complexity of the dynamics, characterized by the number of degrees of freedom, muscular entities, etc. Unfortunately, we do not have access to pinpoint which specific sub-components within the forward dynamics evaluation and integration are most time-consuming. We believe this is a general problem in the field of rigid body dynamics. To improve this, one needs to develop more advanced numerical forward dynamics simulation techniques. #3.5

Minor concerns

Comment 6 :

Missing how the overall muscle length is computed.

We thank the reviewer for their feedback. We provided definitions for both optimal and default muscle fiber lengths in our paper. There might be some confusion regarding the computation of the ratio of muscle fiber length to MTU length. To address this, we have included additional clarification in Fig 5 on how the ratios of muscle fiber length to MTU length are calculated. We hope this enhancement resolves any ambiguities related to the muscle length parameters discussed in our paper. #3.6

Comment 7 :

Model performance under different initial conditions

We thank the reviewer for their suggestion. We have included additional motion comparison analysis with different initial conditions. These initial conditions correspond to different starting poses of the toy musculoskeletal models introduced in the paper. We ran simulations with these different initial conditions for an extended duration of 5 seconds. We kept the rest of the parameters involved, including musculature parameters, joint dynamics, and muscle excitation signals, the same as those used in the paper.

We illustrate the results in Fig R1. As can be seen from the results, large differences between the two methods constantly appear in the Tendon Branch Count and the Joint Count Between Insertion experiments, regardless of various initial conditions. As highlighted in the paper, these differences become more pronounced as the complexity of muscle-tendon branching architectures increases, involving numerous tendon branches and an extensive number of joints spanned by branched tendon insertions. In contrast, the relationship between the motion difference and the ratios of muscle fiber length to MTU length shows a closely linear trend. These additional experiments reinforce the analysis and conclusions made in the manuscript. #3.7

Comment 8 :

Longer simulations to highlight the stability of the proposed methodology

We thank the reviewer for their comment. The set of simulations we ran in response to Comment 7 was extended to five seconds. We hope this shows the stability of the proposed branching modeling method. #3.8

Comment 9 :

Smaller animals such as mice have extremely low masses in the tail and are often difficult to simulate them. Using the proposed approach adds additional complexity with near zeros masses to split the tendons. Is it a limitation of OpenSim or of the formulation?

We thank the reviewer for this insightful question. The current difficulty in modeling musculoskeletal systems with low mass indicates that the limitation primarily stems from the current capabilities of OpenSim. For such small systems, we suggest employing scaling techniques to adjust the mass properties, which will enhance numerical stability and simulation speed, even with more complex branched muscle-tendon architectures. #3.9

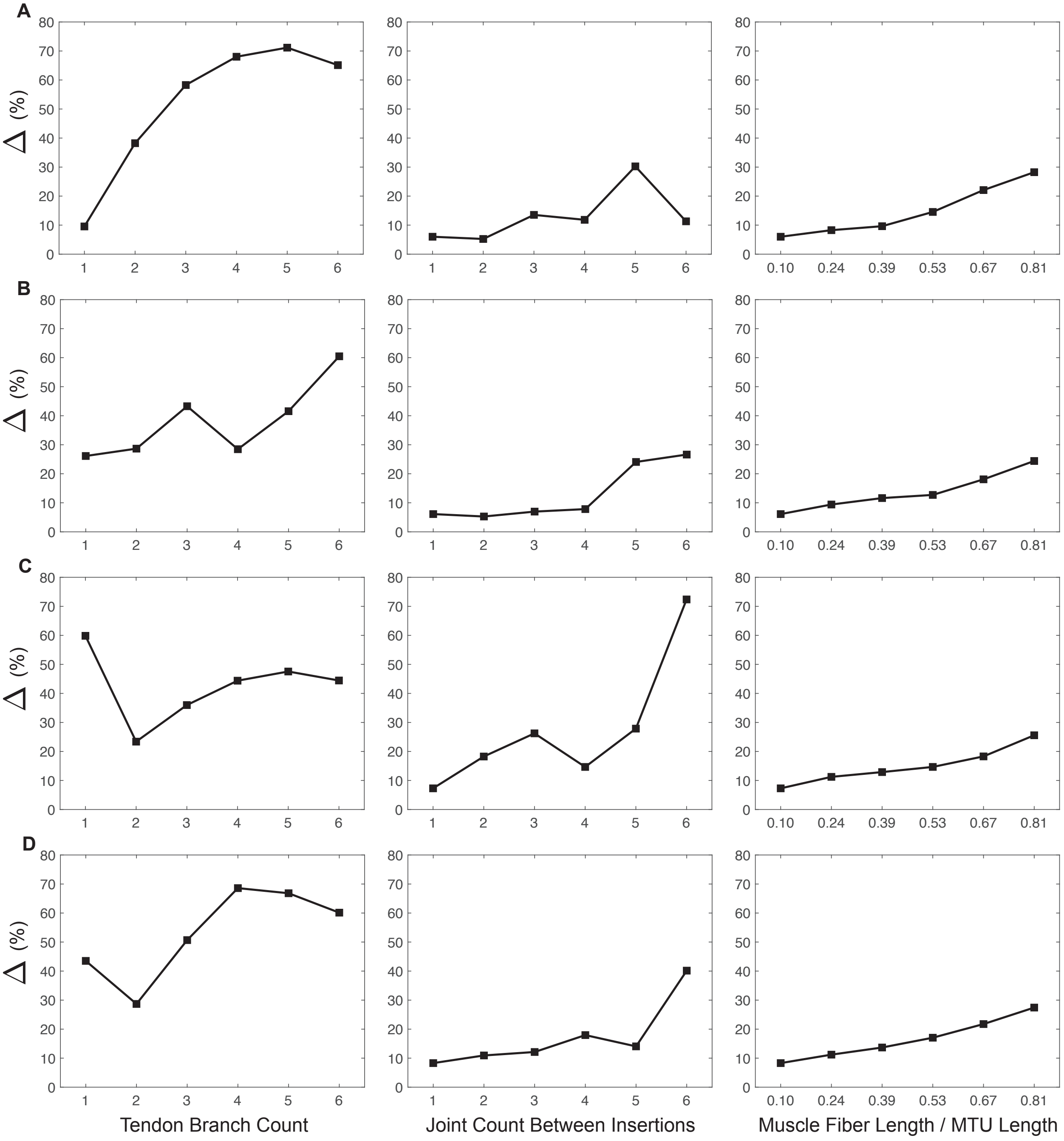

Figure R1: The box plots display the quantitative motion comparison between the proposed branching modeling method and the conventional branching modeling method, evaluated using the same metric as in Fig. 5 of the paper. The four rows, labeled **A**, **B**, **C**, and **D**, correspond to comparative simulations under four different initial pose conditions. Specifically, in **A**, all joint angles start from -20 degrees; in **B**, from -10 degrees; in **C**, from 10 degrees; and in **D**, from 20 degrees. Each row features three columns from left to right, representing three categories of branching muscle-tendon network architectures: varied numbers of tendon branches, joints between insertions, and muscle fiber lengths relative to MTU length ratios, respectively.
